# The Multicentre Acute ischemic stroke imaGIng and Clinical data (MAGIC) repository: rationale and blueprint

**DOI:** 10.3389/fninf.2024.1508161

**Published:** 2025-01-07

**Authors:** Hakim Baazaoui, Stefan T. Engelter, Henrik Gensicke, Lukas S. Enz, Marios Psychogios, Matthias Mutke, Patrik Michel, Davide Strambo, Alexander Salerno, Henk A. Marquering, Paul J. Nederkoorn, Nabila Wali, Stephanie Tanadini-Lang, Björn Menze, Ezequiel de la Rosa, Kaiyuan Yang, Gian Marco De Marchis, Tolga D. Dittrich, Francesco Valletta, Manon Germann, Carlo W. Cereda, João Pedro Marto, Lisa Herzog, Patrick Hirschi, Zsolt Kulcsar, Susanne Wegener

**Affiliations:** ^1^Department of Neurology, University Hospital Zurich, Zurich, Switzerland; ^2^Neurorehabilitation and Neurology, University Department of Geriatric Medicine Felix Platter, University of Basel, Basel, Switzerland; ^3^Department of Neurology and Stroke Center, University of Basel and University Hospital Basel, Basel, Switzerland; ^4^Department of Clinical Research, University of Basel, Basel, Switzerland; ^5^Department of Neuroradiology, University Hospital Basel, Basel, Switzerland; ^6^Stroke Center, Neurology Service, Department of Neurological Sciences, Lausanne University Hospital, University of Lausanne, Lausanne, Switzerland; ^7^Radiology and Nuclear Medicine/Biomedical Engineering and Physics, Amsterdam University Medical Centers, Amsterdam, Netherlands; ^8^Department of Neurology, Amsterdam University Medical Centers, Amsterdam, Netherlands; ^9^Department of Radiation Oncology, University Hospital Zurich, University of Zurich, Zurich, Switzerland; ^10^Department of Quantitative Biomedicine, University of Zurich, Zurich, Switzerland; ^11^Department of Neurology and Stroke Center, Cantonal Hospital St. Gallen, St. Gallen, Switzerland; ^12^DAI di Neuroscienze, Azienda Ospedaliera Universitaria Integrata Verona, Verona, Italy; ^13^Department of Radiology, Cantonal Hospital St. Gallen, St. Gallen, Switzerland; ^14^Stroke Center EOC, Neurocenter of Southern Switzerland, Lugano, Switzerland; ^15^Department of Neurology, Hospital de Egas Moniz, Centro Hospitalar de Lisboa Ocidental, Lisbon, Portugal; ^16^Clinical Data Platform for Research, University Hospital Zurich, Zurich, Switzerland; ^17^Department of Neuroradiology, University Hospital Zurich, Zurich, Switzerland; ^18^Neuroscience Center Zurich, University of Zurich and ETH, Zurich, Switzerland

**Keywords:** ischemic stroke, neuroimaging, data sharing, neurology, collaboration, repository

## Abstract

**Purpose:**

The Multicentre Acute ischemic stroke imaGIng and Clinical data (MAGIC) repository is a collaboration established in 2024 by seven stroke centres in Europe. MAGIC consolidates clinical and radiological data from acute ischemic stroke (AIS) patients who underwent endovascular therapy, intravenous thrombolysis, a combination of both, or conservative management.

**Participants:**

All centres ensure accuracy and completeness of the data. Only patients who did not refuse use of their routine data collected during or after their hospital stay are included in the repository. Approvals or waivers are obtained from the responsible ethics committees before data exchange. A formal data transfer agreement (DTA) is signed by all contributing centres. The centres then share their data, and files are stored centrally on a safe server at the University Hospital Zurich. There, patient identifiers are removed and images are algorithmically de-faced. De-identified structured clinical data are connected to the imaging data by a new identifier. Data are made available to participating centres which have entered into a DTA for stroke research projects.

**Repository setup:**

Initially, MAGIC is set to comprise initial and first follow-up imaging of 2,500 AIS patients. Clinical data consist of a comprehensive set of patient characteristics and routine prehospital metrics, treatment and laboratory variables.

**Outlook:**

Our repository will support research by leveraging the entire range of routinely collected imaging and clinical data. This dataset reflects the current state of practice in stroke patient evaluation and management and will enable researchers to retrospectively study clinically relevant questions outside the scope of randomized controlled clinical trials. New centres are invited to join MAGIC if they meet the requirements outlined here. We aim to reach approximately 10,000 cases by 2026.

## Introduction

Ischemic stroke is a frequent disease and one of the main causes of disability and death in adults worldwide (Goyal et al., 2016; Campbell et al., 2019). Aetiologies, therapy outcomes and short- and long-term functional outcome vary greatly in these patients, rendering individualized therapy concepts highly important (Liebeskind et al., 2017). This requires studies conducted on large, multicentre, high-quality datasets which reflect real world conditions (Bonkhoff and Grefkes, 2022), however, the latter are usually not covered in randomized controlled trials which form the current scientific basis for most treatment decisions.

Recent trends in stroke research include investigating approaches to predict therapeutic success after treatment and functional outcome with or without acute treatment, using both conventional statistical models and machine learning (ML)-based algorithms. This necessitates large, accurate, complete and standardized datasets for more robust predictions (Obermeyer and Emanuel, 2016; Bell and Shimron, 2024; Chen et al., 2024). Current data sharing efforts in the field of stroke evaluation and prognosis have been conducted with several hundred to a few thousand cases (Schirmer et al., 2019; Weaver et al., 2021; Hernandez Petzsche et al., 2022; Liew et al., 2022b; Liu et al., 2023).

In this project, an international academic collaborative team aims to facilitate research addressing current questions in the field of stroke by setting up a collaborative repository between several comprehensive stroke centres, with particular efforts for sharing source imaging data. We therefore established the Multicentre Acute ischemic stroke imaGIng and Clinical data (MAGIC) repository in 2024, initially involving seven stroke centres located at university hospitals in Zurich, Lausanne, Basel, St. Gallen, Lugano, Amsterdam and Lisbon. At the start, MAGIC is set to contain imaging and structured clinical data of approximately 2,500 acute ischemic stroke (AIS) patients in these centres. Given that our initiative features an adaptive design allowing for future inclusion of more centres, we anticipate to reach approximately 10,000 patients within the next 3 years, allowing for advanced ML analyses, e.g., deep learning models which require large datasets to train them (Mouridsen et al., 2020).

The repository includes initial and first follow-up imaging, typically acquired between 1 and 7 days from admission, ranging from computed tomography (CT), CT angiography (CTA) and CT perfusion (CTP) imaging to magnetic resonance imaging (MRI), MR angiography (MRA), MR perfusion weighted imaging (PWI) and digital subtraction angiography (DSA). Clinical data include a pre-specified, comprehensive set of features consisting of patient information and treatment and laboratory variables. New centres are invited to contribute to the repository if they meet the requirements with regards to amount and quality of data, obtain ethics approval and agree to conclude a data transfer agreement (DTA). Planned research applications of the data include advanced imaging studies that correlate imaging features with clinical treatment or outcome variables.

## Goals of the MAGIC repository initiative

The main goal of this initiative is to allow participating centres access to a vast amount of high-quality, multicentre, clinical and imaging data of ischemic stroke patients for conducting research projects, following current European data protection rules. The scope and topics of these projects will be defined on a project level by the respective investigators. We opted for the setup of a data repository rather than a registry because of the relatively lower regulatory hurdles without the need for costly maintenance.

By explaining the intricacies of setting up such a repository of multicentre clinical and imaging data from several countries with different legal frameworks, we strive to provide a blueprint for other groups of investigators with similar aims operating within comparable legal environments. Lastly, we strive to promote our collaboration to potential future participating centres that are willing and able to meet the prerequisites set out in Table 1.

**Table 1:** Prerequisites for each participating MAGIC centre (analogous to Nordanstig et al., 2021 and Scheitz et al., 2018).

MAGIC centres are stroke centres which diagnose and treat patients using state-of-the-art procedures according to current guidelines and systematically document deviations,
are members of the *Endovascular Treatment and Thrombolysis for Ischemic Stroke Patients* (EVA-TRISP) registry,
maintain a prospective registry of consecutive patients with systematic check-up of missing cases,
collect and contribute complete imaging and clinical data (baseline, treatment and follow-up) for initially 300–500 acute ischemic stroke patients,
assess early intracranial haemorrhagic complications and functional outcome at 3 months,
obtain local ethics committee approval (or a waiver for non-applicability), and
enter into a data transfer agreement for data transfer to and from the University Hospital Zurich servers.

## Data

### Included population

The patient population included in the repository consists of all patients that presented with AIS due to large vessel occlusion in the anterior and/or posterior circulation, treated between 2020 and 2023 at the participating centres. All member sites participate in the *Endovascular Treatment and Thrombolysis for Ischemic Stroke Patients* (EVA-TRISP) collaboration, for which principles and responsibilities for joint research initiatives have been defined (Nordanstig et al., 2021). MAGIC consolidates data from AIS patients from the participating centres, including patients who underwent endovascular therapy (EVT), intravenous thrombolysis (IVT), a combination of both, or conservative management. Patients must have undergone acute phase CT and/or MR imaging yielding the diagnosis of vessel occlusion in the suspected ischemic territory, and an assessment of mRS at 3 months is required as a functional outcome measure. Additionally, most of the features (i.e., > 90% in line with the EVA-TRISP requirements (Nordanstig et al., 2021)) detailed in Supplementary material should be available (cf. *Clinical data collection*).

All centres ensure the accuracy and completeness of the provided data. Ethical approvals or waivers are obtained from the respective ethics committees before the data exchange. A formal DTA is negotiated and ratified by all contributing centres. All patient identifiers are removed, and data is transferred via an encrypted file server hosted at the University Hospital Zurich, which allows for transfer of large amounts of data, to be stored centrally at the Zurich site. There, all images are algorithmically de-faced. Upon request, the dataset is made available in bulk to all participating centres for specific projects via the same server. Centres that are not participating in data exchange within the MAGIC consortium can apply for use of data, which requires a collaboration agreement with the participating MAGIC sites. The costs for de-facing and data scrambling are around CHF 1800—per 300 patients while data storage comes in at approximately CHF 500—annually. The former is covered by the individual member sites while the latter is covered by the investigators from the initiating site in Zurich.

### Clinical data collection

After a data sharing agreement between all centres is signed, structured clinical and laboratory features, and imaging data are sent to data protection officers at USZ. Structured clinical data are aggregated and stored in pseudonymized tabular form. For Swiss centres, clinical data is derived from the Swiss Stroke Registry (SSR), (Bonati et al., 2016) where they are contained in structured form and include the EVA-TRISP-specific variables. In the other centres, data is derived from the respective local prospective stroke registry. The collected features include standard clinical information such as age, sex, vascular risk factors, admission laboratory values, acute revascularization treatment, and clinical scores in the acute phase. Functional outcome at 3 months is assessed with the modified Rankin Scale usually performed as a 3-month follow-up, or alternatively by telephone. Furthermore, imaging variables such as collateral scores and revascularization result, measured with the *modified Thrombolysis in Cerebral Infarction* (mTICI) score, are assessed by an imaging core lab at the University Hospital Zurich. Further image features that may be of interest for future projects are assessed on all available images by the sites investigating specific questions. These features are then fed back into the repository for other centres to use. In keeping with current applicable data protection laws and guidelines, patient-identifying features such as name, date of birth, location identifiers and nationality are removed, but not treatment-specific non-identifying timing variables (e.g., onset-to-door delay, door-to-groin delay). Date and time of index event are shifted randomly within a frame of ±3 weeks. Numerical variables are randomly scrambled by a maximum ±5% and the age of patients aged 90 years or older at hospitalization is substituted with “>90 years” to further prevent re-identification. An overview of the included set of clinical variables can be found in Supplementary material.

### Image data collection

Image data contain brain CT, MR and DSA images. The format of choice for transfer of imaging data is *Digital Imaging and Communications in Medicine* (DICOM), as has been customary in medical imaging for decades (Mustra et al., 2008). Once images are pooled, they are converted to the *Neuroimaging Informatics Technology Initiative* (NifTI) format using the *dcm2niix* algorithm (Li et al., 2016). Patient-identifying information from the DICOM file headers are deleted and the original patient identifiers replaced by 16-bit hashes serving as unique project-research identifiers (PRIDs). These PRIDs are generated randomly and allow for coupling of clinical and image data. Whenever conversion to NifTI is not possible due to loss of temporal information, e.g., in the case of CT perfusion or DSA images, identifying header information is still removed. In compliance with local regulations, there is no key allowing for re-identification of patients and therefore, it is not possible to check for additional information in patient files after completion of the data transfer.

**Figure 1:**
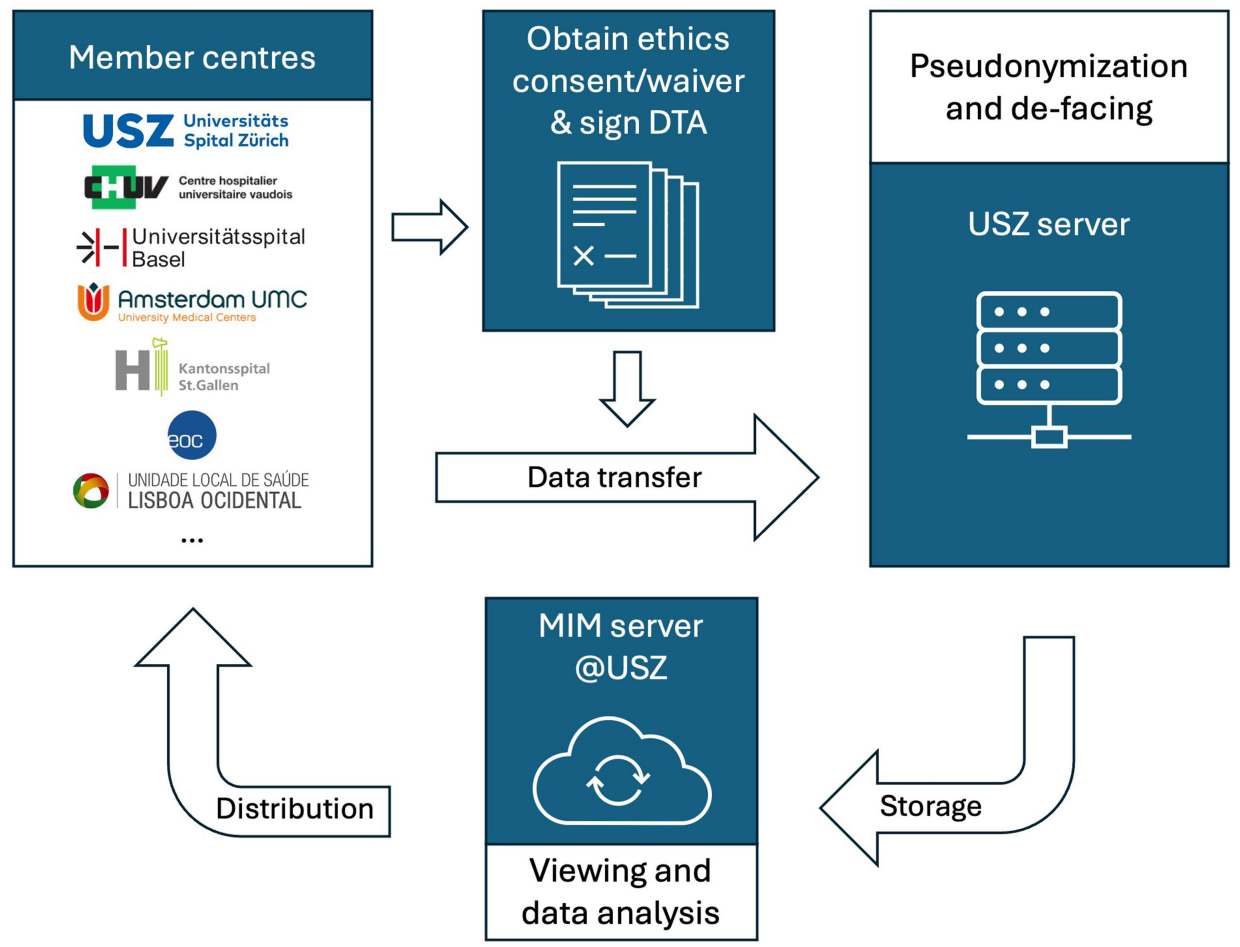
Data flow chart. DTA: data transfer agreement; MIM: Medical Image Merge (software running on the data server); USZ: University Hospital Zurich.

Consequently, proprietary de-facing algorithms are executed on a local machine in *Python* 3.9 (Python Software Foundation, Wilmington, Delaware, United States) to remove identifiable facial features from image data. Scientific imaging processing, analysis and visualisation can be performed on these de-faced NifTI files (Yang et al., 2023). This method of de-identification, recently developed by data engineers at USZ and approved by the local data governance board, has been previously described in detail (Yang et al., 2023). The algorithms will be made available to interested scientific parties upon reasonable request. All imaging data is deposited on a secure server at USZ, where member centres can access them for research projects (cf. below “Anticipated projects”). It is ultimately planned to reconvert the de-faced NifTI images to DICOM format to enable viewing on standard clinical imaging software. An overview of the data transfer process is depicted in Figure 1.

## Ethical and legal considerations

Only patients who did not refuse use of their routine data collected during or after their hospital stay were included in the data transfer (opt-out). Guaranteeing radiological patient data privacy was a main concern, and was considered in two phases: (1) de-identification through removal of DICOM header information and algorithmic de-facing of images, rendering re-identification exceedingly difficult and (2) pseudonymization according to the standards of the National Electronics Manufacturers Association, which maintains the DICOM standard (Robinson, 2014). All information that was not strictly necessary for retaining the usefulness of the data (imaging modality, sequence information) was discarded. Therefore, a common PRID was established for each patient across all data, enabling a connection between images and clinical data.

During the initial planning phase, investigators from USZ submitted a request for “clarification of responsibility” to the responsible ethics committee of the canton of Zurich, including a brief description of the project, its aims and a statement about the intent to publish the findings of studies using the project data. The committee coordinated with the other responsible Swiss ethics committees, namely in Basel and Lausanne. Since the involved committees concluded that the project did not fall within the domain of the Swiss Human Research Act, no ethical approval was required for the Swiss centres and these committees provided a waiver for Swiss centres involved this project. Since maintaining the prospective stroke registry in Amsterdam was subject to ethical approval that had to be renewed, data sharing from Amsterdam was contingent upon successful re-approval. This was obtained before the data transfer. Subsequently joining centres received either approvals or waivers from their responsible local ethics committees.

The legal aspects of data exchange were addressed using a formal DTA, which was drafted by the USZ legal department and then negotiated and signed by the responsible representatives of all participating centres. All signing parties agreed to the stipulations outlined in Table 2, in addition to the usual covenants and indemnifications under Swiss law for any such confidential DTA.

**Table 2:** Requirements set forth in the data transfer agreement.

Use of data exclusively for academic purposes
Storage on a secure server at USZ, where pseudonymization of all data is performed
Access of data solely confined to the group of signing parties
Approval of the local data governance board before distribution of data for individual projects
Avoidance to attempt re-identification of de-identified data
Description of data to be provided, incl. de-identified image and structured clinical data (age, sex, type of cerebrovascular event, location of vessel occlusion, type of stroke treatment, vascular risk factors, admission laboratory and clinical parameters, stroke severity according to the National Institutes of Health Stroke Scale (NIHSS), modified Rankin Scale (mRS) on admission and 3 months after the event, if available from routinely collected clinical records)

## Anticipated projects

Analysis of pseudonymized data can be performed locally at USZ or at the participating study centres. Each centre can also use their own de-identified data for analyses. The scope and topics of potential projects at the member sites, are defined on an individual project level and are at the discretion of the repository members. Due to the joint nature of our repository, collaborative projects can be performed, drawing on the vast experience and knowledge of investigators at the participating centres. In compliance with current regulations, separate proposals to the responsible local data governance boards will be submitted ahead of initiating projects at member sites.

After obtaining access to the secure server at USZ, basic imaging analyses, such as automated region of interest contouring, can be performed natively on the file storage server using the software *MIM* (MIM Software Inc., Cleveland, OH, United States). For advanced applications, e.g., ML-based analyses, centres are granted access on a project basis to download the data to local servers.

Within the EVA-TRISP consortium, several subjects in the field of acute stroke have already been studied using clinical data, including the outcomes of reperfusion therapies after stroke in the anterior cerebral artery territory (Filioglo et al., 2022a), tandem occlusions of the internal carotid artery and anterior cerebral arteries (Filioglo et al., 2022b), and recanalization therapies for large vessel occlusion due to cervical artery dissection (Traenka et al., 2023). With MAGIC, leveraging both the large quantity and high quality of clinical and imaging data, answering clinically relevant questions in the field of stroke, which might be difficult to investigate at a single centre due to insufficient case numbers, is possible.

Suggestions for new projects drawing on repository data can be submitted to the participating centres in the form of a short, informal text document to be approved by the other members. Initiatives can be discussed at “MAGIC meetings,” mostly virtual roundtables of the initiative’s collaborators to discuss the current state of data transfer proceedings and concrete project ideas. We also plan to maintain a file with all ongoing research projects based on the repository data and to discuss the current state and visions for the future of MAGIC during a yearly get-together during a regular stroke conference.

Currently foreseen applications of the MAGIC data include:

- the set-up of open challenges for research groups worldwide with the task to (1) predict the progress of ischemic stroke lesions from acute-phase CT images, as an extension of the *Ischemic Stroke Lesions Segmentation* (“ISLES”) challenges presented at the yearly *International Conference on Medical Image Computing and Computer Assisted Intervention* (“MICCAI”) (Hernandez Petzsche et al., 2022; Liew et al., 2022a; de la Rosa et al., 2024a,b; Riedel et al., 2024), and (2) develop an automated anatomical multiclass segmentation model of the intracranial arterial circulation, which was previously performed for the Circle of Willis (Yang et al., 2023), now to be expanded to more distal vessels,
- an automated leptomeningeal collateral scoring system from DSA and matched CTA data, ultimately used to train ML algorithms for predicting angiographic (mTICI) or functional (NIHSS and mRS) outcomes,
- ML-based classification of stroke aetiology, predicting the likelihood of a cardioembolic aetiology from MR imaging, and
- optimizing the use of admission CT imaging (NCCT and CT perfusion) for early ischemic stroke diagnosis and treatment in patients with ICA/MCA occlusions.

## Discussion

Investigations examining the aetiologies of ischemic stroke (Miceli et al., 2023), relevant factors for the success of reperfusion therapies (Velagapudi et al., 2021; Zhang et al., 2021), the prediction of long-term functional outcome after therapy (Asadi et al., 2014), and further, yet unexplored avenues of advanced stroke research require large datasets including radiology with a high degree of completeness (Bell and Shimron, 2024). MAGIC is a repository aimed at providing participating centres with access to such a collection of ready-to-analyse data by pooling and de-identifying large quantities of medical images and clinical and laboratory features. This blueprint outlines the methodology and structure of MAGIC, with a focus on the administrative, legal, and technical aspects of setting up such a repository.

Our data repository equips affiliated research groups or individual investigators at member institutions with the resources to conduct cutting-edge research without the need for time-consuming project-by-project data collection or having to file and wait for institutional approvals for each individual project. Regulatory hurdles were taken early on to obtain approval from all relevant institutions for conducting research on a pseudonymized, pooled cohort of ischemic stroke patients.

We plan to undertake research projects which leverage the data sharing and collaboration platform that the repository offers. With further members potentially joining our initiative in the future, we expect an increasing number of investigations into specific subjects that benefit from a large, well-curated, multicentre dataset. While this project serves as a “snapshot” multicentre repository, a conversion into a prospective, continuously maintained repository would be a possible future step that the collaborators will assess. Further, after setup of the repository, we will explore making the data available to the scientific community.

## Strengths and limitations

The MAGIC repository boasts various strengths: (1) its comprehensive dataset with the high completeness and amount of variables collected and its large sample size, (2) the focus on most recent data sets which allows to minimize bias from a change over time in stroke treatment standards, (3) the multicentre provenance of data, balancing the influence from each single centre to reduce bias, (4) its collaborative nature with constant and close exchange of the involved parties during the planning, transfer and resulting sub-project phases, incorporating members’ suggestions for an optimized setup, (5) its scalability, allowing new centres to contribute and receive data upon joining the collaboration and becoming a party to the DTA and (6) our platform approach that offers flexibility in terms of use for individual research projects by allowing access to a plethora of de-identified patient data, ready to be used for various studies.

Limitations include: (1) the lack of randomization and comparison groups due to the observational character of the data, rendering exploration of intervention effects difficult. (2) Possible bias could be introduced by only including cases with complete clinical variables and imaging; however, more patients and even incomplete datasets could be incorporated in future projects to explore such bias. (3) Since only angiographically confirmed vessel occlusions are included in this ischemic stroke repository, lacunar infarcts and other non-large vessel occlusion stroke patients will be excluded. This approach was chosen to put together a cohort of strokes with angiographically confirmed vessel occlusion for projects focusing on this problem. However, patients with other stroke characteristics could be included in future data collections. (4) Numerical variables are randomly scrambled by a maximum ±5% to comply with local data protection regulations. We cannot rule out that this may impact the robustness of the data. However, outcome variables are not scrambled, and all other features are left unchanged, thereby limiting the potential impact of data scrambling. (5) Lastly, at the time of establishing the collaboration, member centres were relatively geographically clustered with most data originating from within Switzerland, making it prone to the structural influence of country-wide stroke treatment standards on outcome variables.

## Summary

The MAGIC repository is a data sharing and clinical research collaboration initiated by seven stroke centres situated at university initially in Europe, aimed at consolidating comprehensive data from AIS patients and enabling investigators from participating centres to conduct a breadth of individual or collaborative research projects based on high-quality, pooled multicentre data. The repository will encompass at its onset neuroimaging and thoroughly curated, prospective registry-based structured clinical data of approximately 2,500 AIS patients who underwent EVT, IVT, a combination of both, or conservative management. MAGIC invites participation of more stroke centres willing to meet the requirements outlined above.

## Data Availability

The datasets presented in this article are not readily available because due to Swiss and European data protection laws, only parties that have entered into the data transfer agreement will be granted access to the anonymized data. Requests to access the datasets should be directed to hakim.baazaoui@usz.ch.
