# Supplementary material for: The Multicentre Acute ischemic stroke imaGIng and Clinical data (MAGIC) repository: rationale and blueprint
Source: Front Neuroinform. 2025 Jan 7;18:1508161. doi: 10.3389/fninf.2024.1508161 (PMC11747442; doi:10.3389/fninf.2024.1508161)
Supplement: Supplementary file 1 [file Table_1.docx]

**Supplement.** Features collected in the MAGIC repository*

*subject to availability from each centre

Patient demographics and base information

- Pseudonymized Case ID
- Centre
- Date of arrival at hospital
- Time of arrival at hospital
- Age at time of event
- Sex (M/F)

Medical history

- Stroke (Y/N)
- TIA (Y/N)
- ICH (Y/N)
- Hypertension (Y/N)
- Diabetes (Y/N)
- Hyperlipidemia (Y/N)
- Smoking (Y/N)
- Atrial Fibrillation (Y/N)
- Coronary heart disease (Y/N)
- Prosthetic heart valves (Mechanical/ biological)
- Low ejection fraction (Y/N)
- Peripheral artery disease (Y/N)

Pre-arrival and arrival details

- Prestroke living situation (Home/ nursing home/ rehabilitation hospital/ other medical facility)
- Prestroke disability (modified Rankin Scale)
- Referral (Emergency service (144)/ self referral/ other hospital/ other stroke unit or stroke centre/ general practitioner)
- Transport (Ambulance/ helicopter/ other)

Clinical presentation and initial assessment

- Stroke syndrome (Bamford classification)
- NIHSS on admission
- GCS on admission
- 1st systolic blood pressure (mmHg)
- 1st diastolic blood pressure (mmHg)
- Height (cm)
- Weight (kg)
- BMI (kg/m²)
- Time of symptom onset known (Y/N)
- Onset date
- Onset time
- Wake-up date
- Wake-up time
- Last known well date
- Last known well time

Pre-stroke medications

- Aspirin pre-stroke (Y/N)
- Clopidogrel pre-stroke (Y/N)
- Prasugrel pre-stroke (Y/N)
- Ticagrelor pre-stroke (Y/N)
- Dipyridamole pre-stroke (Y/N)
- Vit. K ag pre-stroke (Y/N)
- Vit. K ag INR (Y/N)
- Rivaroxaban pre-stroke (Y/N)
- Dabigatran pre-stroke (Y/N)
- Apixaban pre-stroke (Y/N)
- Edoxaban pre-stroke (Y/N)
- Parenteral anticoagulation pre-stroke (Y/N)
- Antihypertensive drugs pre-stroke (Y/N)
- Lipid-lowering drugs pre-stroke (Y/N)
- Hormone replacement or contraception (Y/N)

Acute medications and treatment

- Antiplatelet drugs administered (Y/N)
- Anticoagulants administered (Y/N)
- IVT with rtPA (Y/N)
- IVT start date
- IVT start time
- Total rtPA dose (mg)
- IAT (Y/N)
- Date of groin puncture
- Time of groin puncture
- IAT end date
- IAT end time
- Anesthesia (General anesthesia/ conscious sedation)
- IAT rtPA (Y/N)
- IAT rtPA dose (mg)
- IAT urokinase (Y/N)
- IAT urokinase dose (mg)
- IAT mechanical treatment (Y/N)
- IAT stent retriever (Y/N)
- IAT aspiration (Y/N)
- IAT distal retriever (Y/N)
- IAT balloon (Y/N)
- IAT intracranial stent (Y/N)
- IAT extracranial stent (Y/N)
- IAT other mechanical (Y/N)

Timing variables

- Door to image (min.)
- Onset to treatment (min.)
- Door to treatment (min.)
- Onset to groin puncture (min.)
- Door to groin puncture (min.)

Imaging and diagnostic tests

- 1st brain imaging type
- 1st brain imaging date
- 1st brain imaging time
- 1st brain imaging result
- Acute perfusion imaging type
- Acute perfusion imaging result
- 1st vascular imaging type
- 1st vascular imaging result
- Follow-up brain imaging type
- Follow-up brain imaging result
- Follow-up MR-angiography (Y/N)
- Follow-up CT-angiography (Y/N)
- Follow-up Ultrasound (Y/N)
- Follow-up Digital subtraction angiography (Y/N)
- Follow-up Transthoracic echo (Y/N)
- Follow-up Transesophageal echo (Y/N)
- Follow-up Holter ECG (Y/N)
- Follow-up Holter days

Laboratory results

- 1st glucose (mmol/l)
- 1st cholesterol total (mmol/l)
- 1st cholesterol LDL (mmol/l)
- 1st creatinine (µmol/l)

Outcomes and follow-up

- NIHSS 24h
- Symptomatic ICH (Y/N)
- Symptomatic ICH date
- Recurrent stroke (Y/N)
- Recurrent stroke date
- Orolingual angioedema (Y/N)
- Death in hospital (Y/N)
- Death in hospital date
- Death in hospital time
- Death in hospital cause
- Epileptic seizure in hospital (Y/N)
- Epileptic seizure in hospital date
- Decompressive craniectomy (Y/N)
- Decompressive craniectomy date
- Carotid endarterectomy (Y/N)
- Carotid endarterectomy date
- Carotid artery stenting (Y/N)
- Carotid artery stenting date
- Other endovascular revascularization (Y/N)
- Other surgical revascularization (Y/N)
- Other surgical revascularization date
- Other surgical revascularization specification (free text)
- PFO closure (Y/N)
- PFO closure date
- Discharge destination
- Discharge date
- Discharge time
- Discharge mRS
- Duration of hospital stay (days)
- 3M date
- 3M mRS
- 3M NIHSS
- 3M Stroke (Y/N)
- 3M Stroke date
- 3M ICH (Y/N)
- 3M ICH date
- 3M Death (Y/N)
- 3M Death date
- 3M Death cause
- 3M Epileptic seizure (Y/N)
- 3M Epileptic seizure date
